# Dynamic Contrast-Enhanced MRI in Neuro-Oncology: A Narrative and Translational Review

**DOI:** 10.3390/brainsci16090991

**Published:** 2026-09-19

**Authors:** Francesca Bozzetti, Ana Ramos-González, Alessandro Leonetti, Chiara Tommasi, Marcello Tiseo, Francesco Salaroli, Giovanni Ceccon, Ilaria Renna, Marco Galaverni, Nicola Sverzellati, Daniele Corbo

**Affiliations:** 1 Neuroradiology Unit, Diagnostic Department, University Hospital of Parma, 43121 Parma, Italy; 2 Department of Medicine and Surgery, University of Parma, 43121 Parma, Italy; mtiseo@ao.pr.it (M.T.);; 3 Department of Neuroradiology, University Hospital Madrid, 28015 Madrid, Spain; 3anaramos@gmail.com; 4 Medical Oncology Unit, University Hospital of Parma, 43121 Parma, Italy; aleonetti@ao.pr.it; 5 Medical Oncology and Breast Unit, University Hospital of Parma, 43121 Parma, Italy; 6 Radiotherapy Unit, University Hospital of Parma, 43121 Parma, Italy; fsalaroli@ao.pr.it (F.S.); irenna@ao.pr.it (I.R.);; 7 Unit of Radiological Sciences, Diagnostic Department, University Hospital of Parma, 43121 Parma, Italy; 8 Department of Medical and Surgical Specialties, Radiological Sciences and Public Health, University of Brescia, 25123 Brescia, Italy

**Keywords:** DCE-MRI, tumor, MRI, neuro-oncology

## Abstract

Background/Objectives: Dynamic contrast-enhanced MRI (DCE-MRI) is emerging as a key technique in neuro-oncologic imaging by enabling quantitative assessment of vascular permeability and blood–brain barrier integrity, two central features of tumor progression and treatment-related tissue injury. Its clinical relevance is particularly evident in the post-treatment setting, where conventional MRI lacks specificity and entities such as tumor progression, pseudoprogression, and radiation necrosis frequently overlap. Methods: This narrative review focuses on the role of DCE-MRI in differentiating treatment-related alterations from true tumor progression, primarily in gliomas and, secondarily, in brain metastases and primary CNS lymphoma. Results: We review quantitative and semiquantitative parameters, their biological interpretation, and their diagnostic performance in gliomas, metastases, and primary CNS lymphoma. We further discuss the role of DCE-MRI within multiparametric imaging workflows and highlight emerging contributions from artificial intelligence, radiomics, and automated pharmacokinetic modeling. Conclusion: As a narrative, translational review, this work aims to contextualize DCE-MRI within contemporary neuro-oncologic practice and research. Its clinical translation, however, remains limited by methodological heterogeneity and the lack of standardized acquisition and analysis protocols, highlighting the need for harmonization and prospective multicenter validation.

## 1. Introduction

Brain tumors are biologically heterogeneous diseases whose growth, invasiveness, and response to therapy are closely related to angiogenesis, vascular permeability, and blood–brain barrier disruption. Although contrast-enhanced MRI remains the cornerstone of neuro-oncologic imaging, conventional enhancement patterns lack specificity, particularly after treatment. In this setting, tumor progression, pseudoprogression, radiation necrosis, and other treatment-related alterations may show overlapping imaging features, leading to diagnostic uncertainty and potentially inappropriate clinical decisions. DCE-MRI provides quantitative and semi-quantitative information on contrast agent kinetics, offering indirect but biologically meaningful markers of microvascular permeability and blood–brain barrier integrity. These features make DCE-MRI particularly valuable for interpreting contrast enhancement in relation to underlying tumor biology and treatment-induced vascular damage. Despite its conceptual appeal, DCE-MRI remains one of the most technically demanding perfusion techniques in neuro-oncology. Quantitative permeability metrics such as Ktrans, Ve, and Kep are highly sensitive to acquisition parameters, arterial input function estimation, model selection, and post-processing pipelines. As a result, reproducibility across scanners, institutions, and even repeated examinations in the same patient is often limited. This lack of standardization has led many clinical centers to move away from strict quantitative thresholds and instead adopt qualitative or semi-quantitative curve-based interpretation. Acknowledging these constraints is essential for framing the translational potential of DCE-MRI and for understanding why its clinical adoption remains uneven despite strong biological foundations.

This review aims to summarize the evolving role of DCE-MRI in neuro-oncology, focusing on its translational value from vascular biology to clinical decision-making. Particular emphasis is placed on its application in the post-treatment assessment of gliomas and brain metastases, where differentiating true progression from treatment-related changes remains a major clinical challenge. Additional applications in primary CNS lymphoma, multiparametric imaging frameworks, and artificial intelligence-based approaches are also discussed, together with current limitations and future directions toward standardized, reproducible, and clinically applicable DCE-MRI biomarkers.

### 1.1. Technical Principles and Interpretation of DCE-MRI Parameters

Dynamic contrast-enhanced MRI characterizes tissue microvascular properties by acquiring serial T1-weighted images before, during, and after intravenous administration of a gadolinium-based contrast agent. Signal-intensity changes are converted into contrast-agent concentration–time curves and analyzed using pharmacokinetic models. The most widely used approach in neuro-oncology is the Tofts model, which describes contrast-agent exchange between the blood plasma and the extravascular extra-cellular space (EES). The standard Tofts model assumes that the contribution of the intravascular plasma compartment to the measured tissue concentration is negligible, whereas the extended Tofts model incorporates an additional plasma-volume term (Vp), which may be relevant in highly vascular tumors. The principal quantitative DCE-MRI parameters are Ktrans, Kep, fractional volume of the EES (Ve), and Vp. Ktrans (min^−1^) is the volume transfer constant describing contrast-agent transfer from blood plasma into the EES. Although frequently interpreted as a marker of vascular permeability, Ktrans is influenced by both permeability and blood flow and therefore should not be regarded as a direct or isolated measurement of blood–brain barrier permeability. Its physiological meaning depends on the underlying flow–permeability relationship and on the pharmacokinetic model applied [1,2]. Ve (dimensionless, usually expressed as a fraction between 0 and 1) represents the fractional volume of the EES. It may provide information about extracellular space characteristics but is sensitive to model fitting and may become unstable when enhancement is limited or when model assumptions are violated. Kep (min^−1^) represents the rate constant describing contrast-agent reflux from the EES to the plasma and is mathematically related to Ktrans and Ve according to the relationship Kep = Ktrans/Ve. Consequently, Kep is not an independent physiological measurement and inherits uncertainties associated with both Ktrans and Ve. Vp (dimensionless fractional plasma volume) estimates the fraction of tissue volume occupied by blood plasma and is explicitly modeled in the extended Tofts approach. Although it may provide information related to vascular volume, it should not be considered interchangeable with blood-volume parameters derived from dynamic susceptibility contrast (DSC) MRI. In addition to pharmacokinetic parameters, the initial area under the concentration–time curve (iAUC) is a model-independent or semiquantitative metric obtained by integrating the contrast-agent concentration curve over a predefined early time interval after injection. Depending on implementation, iAUC may be reported as concentration × time or as a normalized/arbitrary value. It reflects the combined effects of blood flow, vascular volume, permeability, and extracellular distribution and therefore cannot be interpreted as a specific measure of any single physiological process. The biological interpretation of all DCE-derived parameters requires caution. Quantitative estimates depend on several methodological factors, including scanner platform and magnetic field strength, temporal and spatial resolution, pre-contrast T1 measurement, contrast-agent dose and injection rate, conversion of signal intensity to contrast concentration, selection or estimation of the arterial input function, pharmacokinetic model selection, and post-processing implementation [1,3]. Moreover, the assumptions underlying compartmental models may not be fully satisfied in heterogeneous tumors or treatment-related lesions, where necrosis, hemorrhage, abnormal vascular architecture, and spatially variable blood–brain barrier disruption may coexist. Accordingly, absolute thresholds reported by individual studies should not automatically be transferred across scanners, acquisition protocols, or post-processing platforms. These limitations are central to understanding why quantitative DCE-MRI remains difficult to standardize and why semiquantitative and multiparametric approaches continue to play an important role in clinical neuro-oncology. Wider clinical implementation will require harmonized acquisition and post-processing protocols, standardized parameter definitions and reporting, and prospective multicenter validation in sufficiently large and clinically representative cohorts [1,3].

### 1.2. Role of DCE-MRI in Assessing Vascular Permeability and Contrast Dynamics

DCE-MRI provides quantitative characterization of lesion vascular permeability through parameters such as Ktrans and Kep, which serve as indirect markers of microvascular permeability and blood–brain barrier disruption [1]. DCE-derived parameters are closely linked to the appearance of contrast enhancement on conventional MRI sequences. The degree and pattern of enhancement are strongly influenced by vascular permeability: lesions with low permeability tend to show greater signal intensity on contrast-enhanced T2 FLAIR sequences, whereas highly permeable lesions are more conspicuous on contrast-enhanced T1-weighted imaging. These differences reflect contrast dynamics, with increased permeability leading to greater accumulation of contrast agent within the lesion and sequence-dependent signal modulation. Quantitative analyses demonstrate a consistent relationship between DCE parameters and imaging appearance, with CE-T2 FLAIR signal intensity inversely correlated with permeability measures such as Ktrans and Kep. These findings support a physiologically grounded interpretation of contrast enhancement, linking imaging features to microvascular function and BBB integrity. Relative enhancement patterns across sequences may therefore serve as indirect indicators of tumor permeability, with potential implications for treatment planning and drug delivery. Wang et al. 2023 [2] investigated water exchange across the BBB as an emerging biomarker of BBB dysfunction in high-grade gliomas, comparing DCE-MRI with vascular water exchange imaging (VEXI). A significant correlation was observed between DCE-derived parameters and VEXI metrics, suggesting that both techniques capture related physiological processes linked to BBB permeability and water exchange.

This comparability indicates that DCE-MRI, given its wider availability, may serve as a practical surrogate for more advanced techniques in the assessment of BBB dysfunction. However, the exploratory design and limited sample size require further validation to confirm reproducibility and clinical applicability.

Overall, these findings support the expanding role of DCE-MRI in neuro-oncologic imaging, highlighting its ability to assess not only vascular permeability but also broader aspects of BBB physiology within a multiparametric framework.

**Figure 1:**
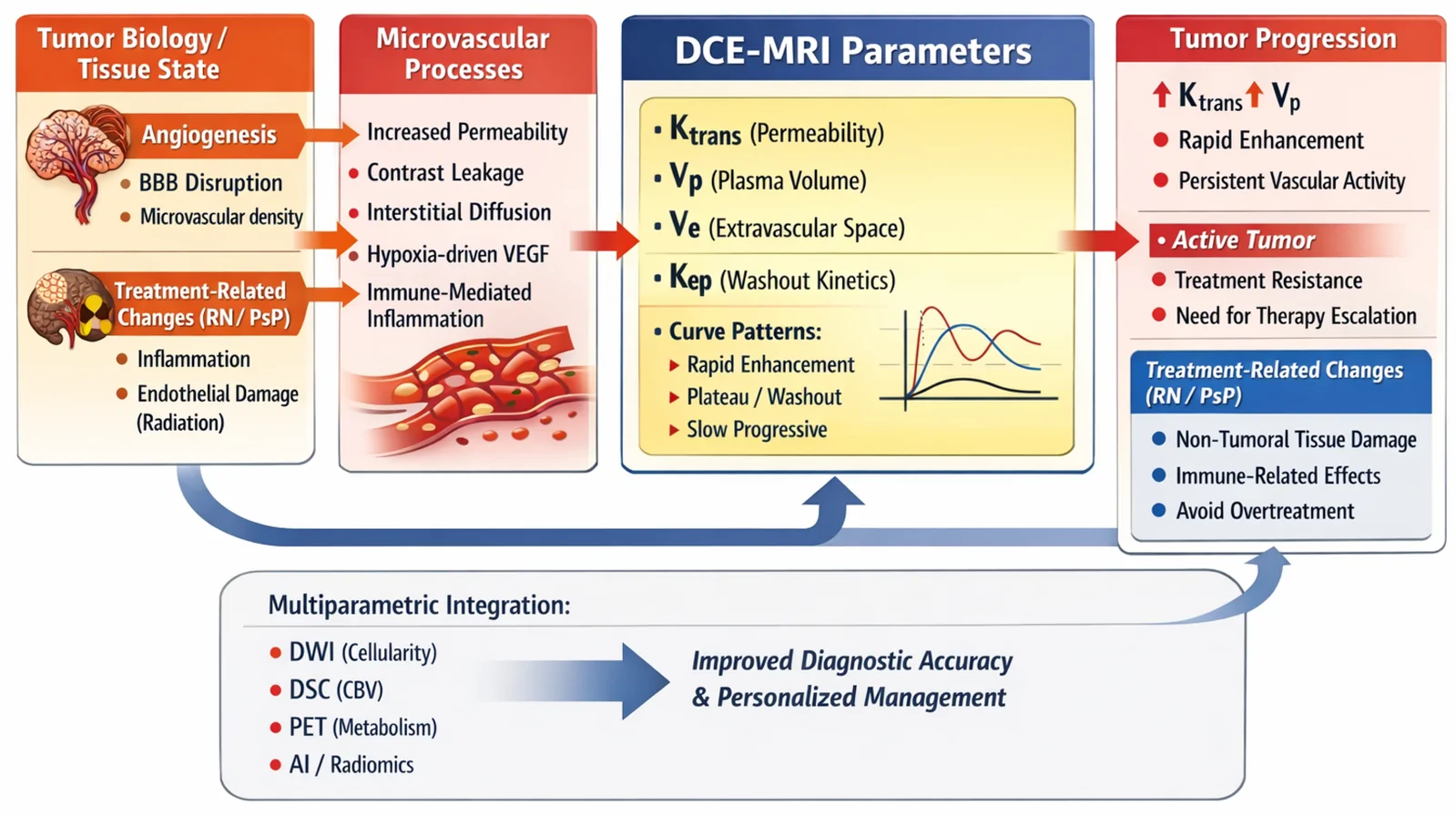
A conceptual overview illustrating how DCE-MRI quantitatively links tumor biology, microvascular permeability, and treatment-related tissue changes to clinically relevant parameters. The diagram highlights how contrast-agent kinetics reflect underlying processes such as angiogenesis, BBB disruption, inflammation, and hypoxia, and how multiparametric integration with DWI, DSC, PET, and radiomics enhances diagnostic accuracy and supports personalized neuro-oncology decision-making.

DCE-MRI can be conceptually interpreted as a translational bridge linking microvascular biology to clinical decision-making, as illustrated in Figure 1.

## 2. Treatment-Related Alterations

Treatment-related alterations (TRAs) encompass biologically distinct entities with different temporal evolution and clinical implications. Pseudoprogression and radiation necrosis are particularly challenging because their imaging appearance may overlap with tumor progression on conventional MRI. Pseudoresponse represents a different treatment-related phenomenon, typically associated with anti-angiogenic therapy, in which a rapid reduction in contrast enhancement reflects decreased vascular permeability rather than a true reduction in tumor burden. Given these overlapping imaging appearances, advanced imaging biomarkers may provide complementary information for distinguishing treatment-related effects from a viable tumor. The key biological features, DCE-MRI patterns, and clinical implications of pseudoprogression, radiation necrosis, and tumor progression are summarized in Table 1 [3].

## 3. Treatment-Related Alterations vs. Tumor Progression

Biological mechanisms, DCE-MRI permeability patterns, complementary imaging features, and clinical implications for pseudoprogression, radiation necrosis, and true tumor progression, high-lighting how vascular injury, inflammation, and active angiogenesis shape diagnostic interpreta-tion in the post-treatment setting are summarized in Table 1.

### 3.1. Pseudoprogression

Pseudoprogression (PsP) represents a subacute treatment-related effect reflecting transient blood–brain barrier disruption and inflammatory changes. A retrospective study in glioblastoma reported an incidence of approximately 29%, consistent with previously described rates of 20–30%. Most cases occurred beyond four months after chemoradiotherapy, suggesting that delayed PsP may be more common than traditionally assumed. Importantly, the temporal distribution of pseudoprogression is not confined to a rigid post-treatment window. Although PsP is classically regarded as an early treatment-related phenomenon, later presentations are increasingly recognized, and the timing of onset may vary according to the therapeutic context. Therefore, early and delayed PsP should be considered as overlapping temporal patterns rather than mutually exclusive categories, and timing alone should not be used to distinguish PsP from true tumor progression. From a prognostic perspective, PsP was associated with improved overall survival compared to true progression, supporting its interpretation as a marker of treatment response. Patients with late PsP showed better outcomes than those with early PsP, likely reflecting higher completion rates of standard chemoradiotherapy protocols, a key determinant of survival [4]. Clinical and treatment-related factors showed limited and inconsistent associations. Male sex and completion of chemoradiotherapy were associated with PsP in some analyses, whereas no clear relationships were found with performance status or type of surgical intervention. Regarding molecular markers, no significant correlation was observed between PsP and MGMT promoter methylation, contrary to previous reports, possibly due to variability in assessment methods and thresholds. Similarly, no consistent associations were identified with common genomic alterations, including TP53, PTEN, EGFR, and TERT. The study is limited by its retrospective design, potential selection bias, and incomplete data, including missing genomic information and variability in imaging assessment. The lack of standardized use of advanced imaging techniques, such as perfusion MRI, may have affected differentiation between PsP and true progression. In addition, the definition of PsP is inherently retrospective, requiring longitudinal imaging stability and potentially delaying clinical decision-making. Overall, these findings reinforce the clinical relevance of PsP as a favorable prognostic indicator and highlight the need for standardized molecular and imaging criteria. Integration of advanced imaging modalities, including perfusion MRI, MR spectroscopy, and amino acid PET, may enable more accurate and earlier differentiation between PsP and true tumor progression. Immune checkpoint inhibitors (ICIs) are increasingly used in the treatment of systemic cancers, particularly lung cancer and melanoma, and have demonstrated efficacy in brain metastases due to their ability to cross the blood–brain barrier. However, PsP in this setting remains poorly characterized, with current evidence limited to small series and case reports [5]. According to iRANO criteria, PsP is defined as radiological worsening within the first six months of ICI therapy in the absence of clinical deterioration, followed by subsequent stability or improvement. In this cohort, however, clinical worsening was not considered an absolute exclusion criterion, as neurological symptoms may result from mass effect rather than true tumor progression. A subset of symptomatic patients treated with corticosteroids showed both clinical improvement and radiological stabilization, indicating that PsP may occur even in the presence of symptoms and should be assessed on a case-by-case basis. Biologically, PsP reflects treatment-induced immune activation, leading to inflammatory infiltration and transient lesion enlargement. This effect may be more pronounced with combination immunotherapy (e.g., nivolumab plus ipilimumab), which is associated with stronger immune responses and a higher incidence of immune-related adverse events. Consistently, higher PD-L1 expression was observed in the PsP group, supporting its role as a potential biomarker of enhanced immune activation. Greater tumor burden was also associated with PsP, although this finding requires further validation. Patients with PsP showed a trend toward improved survival, consistent with its interpretation as a favorable prognostic marker. Importantly, early radiological worsening may lead to premature discontinuation of effective therapy or unnecessary additional treatments, underscoring the need for careful interpretation.

### 3.2. Radiation Necrosis

Radiation necrosis (RN) is a delayed complication of radiotherapy, with an incidence generally ranging from approximately 5% to 25%, depending on treatment parameters, follow-up duration, and patient population. It typically develops within 3 to 40 months after radiotherapy. Notably, in a study based on histopathologically confirmed cases, Hazaymeh [6] reported a lower incidence of approximately 5% among patients with suspected tumor recurrence, highlighting the potential overestimation of RN when diagnosis relies solely on imaging findings. From a biological perspective, RN results from radiation-induced neurovascular injury, involving endothelial and glial cell damage within irradiated brain tissue. This process leads to fibrinoid necrosis, microvascular thrombosis, and local ischemia, followed by hypoxia-driven activation of microglia and upregulation of hypoxia-inducible factor-1α (HIF-1α), which promotes VEGF expression and results in abnormal, leaky angiogenesis, vasogenic edema, and a self-sustaining inflammatory cascade [7]. Clinically, RN typically manifests months to years after radiotherapy and presents with a highly variable spectrum, ranging from asymptomatic imaging findings to significant neurological impairment, including cognitive decline, focal deficits, and seizures. However, a substantial proportion of cases remain clinically silent or exhibit a relatively indolent course [6]. This variability, together with the substantial overlap of imaging features with tumor recurrence, makes accurate diagnosis particularly challenging and may directly impact clinical management, potentially leading to unnecessary interventions or delayed treatment. The development of RN is influenced by multiple risk factors, predominantly related to radiotherapy parameters, including total dose, fractionation scheme, irradiated brain volume, and cumulative exposure, particularly in re-irradiation settings [8]. The risk is higher with stereotactic radiosurgery (SRS), especially when delivered in a single fraction and in larger lesions, whereas multifraction SRS appears to mitigate this risk. Additional contributing factors include advanced age, prior radiation exposure, and concomitant systemic therapies, including chemotherapy and immunotherapy [9]. Tumor biology and location may also influence susceptibility, with higher incidence reported in specific molecular subtypes and anatomically vulnerable regions such as periventricular and infratentorial areas [7].

## 4. Clinical Applications of DCE-MRI in Neuro-Oncology

Table 2 Summarizes the main studies on DCE-MRI in glioblastoma, highlighting key imaging parameters, principal findings, and clinical contributions.

Across recent investigations, DCE-MRI has shown meaningful potential for distinguishing true tumor progression from treatment-related changes in gliomas, yet the collective evidence also illustrates how technically demanding and variable the technique remains in practice. Semiquantitative approaches appear particularly useful in the early post-treatment phase, when conventional MRI is least reliable. Vankan et al. [10] demonstrated that peak enhancement percentage can separate progression from treatment-related inflammation with high accuracy, showing lower values in treatment-related changes and rapid, high-amplitude enhancement in progressive tumors. Their kinetic observations reinforce the biological distinction between necrotic, low-permeability tissue and actively angiogenic tumor, and highlight how curve morphology can support interpretation when pharmacokinetic modeling is impractical. Var et al. [11] extended this perspective by combining GRASP-based DCE-MRI with DSC-MRI, showing that permeability metrics such as Ktrans, Vp, and Ve add diagnostic value to rCBV, especially at the first clinical suspicion of progression. The benefit was strongest early after therapy and diminished over time, suggesting that multiparametric imaging may be most informative when diagnostic uncertainty is highest. Their work also underscored the importance of longitudinal assessment, as progressive disease tended to show increasing perfusion and permeability over successive scans.

Other studies have focused on regions that conventional imaging often fails to characterize adequately. Diao et al. [12] examined the non-enhancing peritumoral region, demonstrating that combining DCE-MRI with diffusion tensor imaging can reveal infiltrative tumor components that would otherwise be indistinguishable from treatment-related edema. Increased permeability and reduced microstructural integrity were consistent markers of progression, and integrating vascular and diffusion features markedly improved diagnostic performance. Cao et al. [13] approached the same problem from a different angle, using histogram-based analysis to capture intratumoral heterogeneity. Their results showed that spatially distributed permeability metrics can predict progression months before it becomes radiologically evident, emphasizing the value of methods that move beyond simple mean-value measurements. More recent work by Wang et al. [14] and Zhu et al. [15] has taken this concept further by applying voxel-wise clustering to DCE and diffusion parameters, identifying biologically meaningful tumor habitats with distinct combinations of cellularity, vascular permeability, and hypoxia. These spatially resolved phenotypes were associated with IDH status, treatment resistance, and survival, suggesting that DCE-MRI can contribute to a more nuanced understanding of tumor microenvironmental architecture.

Finally, Zakhari et al. [16] addressed a practical issue that resonates strongly with clinical experience: the variability in qualitative interpretation. Their findings showed that visual assessment of enhancement patterns and diffusion restriction suffers from substantial interobserver disagreement, whereas quantitative perfusion parameters—including AUC, Vp, and Ktrans—demonstrate more consistent reproducibility. Even so, the reliability of these metrics depends heavily on acquisition protocols, modeling choices, and reader expertise, reinforcing the need for standardization before quantitative DCE-MRI can be considered fully actionable. Taken together, these studies illustrate both the promise and the limitations of DCE-MRI. The technique provides biologically grounded markers of vascular permeability and microenvironmental complexity, and its integration with diffusion imaging or DSC-MRI can substantially improve diagnostic confidence. At the same time, variability across scanners, protocols, and modeling approaches continues to limit reproducibility, explaining why many centers rely primarily on qualitative curve analysis or multiparametric frameworks rather than strict pharmacokinetic quantification. The evidence suggests that DCE-MRI is most valuable when used as part of an integrated strategy, particularly in early or diagnostically uncertain cases, while also highlighting the need for broader standardization and prospective validation.

## 5. Translational Interpretation of DCE-MRI in Glioblastoma

Glioblastoma is characterized by marked biological heterogeneity, driven by complex interactions between tumor proliferation, angiogenesis, and microenvironmental factors such as hypoxia and blood–brain barrier disruption. These processes result in highly variable vascular permeability and spatially heterogeneous tumor compartments, which are only partially captured by conventional imaging. Within this context, DCE-MRI provides quantitative assessment of vascular permeability through parameters such as Ktrans, offering a biologically grounded marker of tumor angiogenic activity and blood–brain barrier integrity. Increased permeability is generally associated with active tumor progression, reflecting neovascularization and microvascular dysfunction, whereas treatment-related changes—such as pseudoprogression and radiation necrosis—tend to show lower or heterogeneous permeability patterns related to inflammation, endothelial damage, and tissue necrosis. From a clinical perspective, this biological–imaging relationship is particularly relevant in the post-treatment setting, where differentiating tumor progression from treatment-related alterations remains a major challenge. Evidence from the reviewed studies indicates that DCE-MRI improves diagnostic accuracy, especially when integrated with complementary techniques such as diffusion and DSC perfusion imaging, allowing for a multiparametric characterization of tumor behavior. In addition, advanced analytical approaches, including histogram analysis, habitat imaging, and artificial intelligence, highlight the importance of capturing intratumoral heterogeneity beyond mean-value metrics. Overall, DCE-MRI may contribute to bridging tumor biology and clinical decision-making in glioblastoma, although its full clinical implementation requires standardization of acquisition protocols, parameter thresholds, and analytical methods.

## 6. Metastasis

Advanced MRI techniques provide a multiparametric framework for the characterization of tumor biology, integrating information on perfusion, vascular permeability, and cellularity. An overview of the main studies on DCE-MRI in brain metastases is provided in Table 3, allowing for rapid identification of key findings and clinical applications.

In this context, DCE-MRI enables quantification of microvascular properties through parameters such as Ktrans, Ve, and Vp, reflecting blood–brain barrier permeability and microvascular density. Semiquantitative metrics, including peak enhancement and area under the curve (AUC), as well as time–intensity curve patterns, further describe contrast dynamics. Complementarily, DWI provides information on tissue microstructure, with ADC values reflecting cellularity, where lower ADC values are associated with higher tumor cell density and aggressiveness. In the post-treatment setting, particularly in brain metastases, differentiating radiation-induced changes, including radiation necrosis, from tumor recurrence remains a major challenge. Multiparametric MRI approaches combining DWI, DCE, DSC, and arterial spin labeling (ASL) capture complementary aspects of tumor biology and improve diagnostic assessment. From a pathophysiological perspective, treatment-related changes are typically associated with increased ADC values, reflecting apoptosis and reduced cellularity, whereas tumor recurrence shows lower ADC due to increased proliferation. Similarly, reductions in CBV and Ktrans over time suggest post-radiation effects, while elevated rCBV and Ktrans are more consistent with recurrent tumor, reflecting active neovascularization and persistent blood–brain barrier disruption. DCE- and DSC-derived parameters demonstrate strong diagnostic performance, with high sensitivity and specificity for Ktrans and rCBV in identifying recurrence. However, overlap between post-treatment changes and tumor progression, along with variability in acquisition and analysis methods, limits the reliability of individual parameters when used in isolation [17]. Brain metastases are biologically heterogeneous lesions whose growth and progression are closely linked to angiogenesis, vascular permeability, and BBB disruption. Tumor proliferation is associated with active neovascularization, increased microvascular density, and enhanced permeability, facilitating contrast agent leakage and reflecting an aggressive microenvironment. In contrast, treatment-related effects, such as radiation-induced changes and radionecrosis, are characterized by endothelial damage, inflammation, and microvascular injury, often resulting in reduced perfusion and altered permeability patterns. Within this framework, DCE-MRI provides quantitative assessment of vascular permeability, offering physiologically relevant biomarkers that link imaging features to underlying tumor biology and microenvironmental dynamics. From a clinical perspective, the management of brain metastases requires both accurate lesion detection and reliable differentiation between tumor recurrence and treatment-related effects during follow-up. Although contrast-enhanced MRI remains the standard modality, its specificity is limited in the post-treatment setting, particularly after SRS, where overlapping imaging features may lead to diagnostic uncertainty and inappropriate therapeutic decisions. Advanced imaging techniques have therefore emerged as complementary tools. DWI provides information on cellularity, MR spectroscopy assesses metabolic alterations, and PET, particularly with amino acid tracers, evaluates tumor metabolism. Among these modalities, DCE-MRI provides complementary quantitative information on contrast-agent kinetics and microvascular permeability, which may support the detection and characterization of treatment-induced vascular changes and improve differentiation between viable tumor and radiation-related effects. The integration of multiparametric imaging approaches supports a more comprehensive and biologically informed evaluation of brain metastases, potentially improving diagnostic accuracy and contributing to more personalized patient management [18].

Azab et al. [20] provided a systematic review on the role of perfusion MRI in the evaluation of brain metastases, highlighting the limitations of conventional imaging in lesion characterization and treatment monitoring. The review demonstrated that perfusion-based MRI techniques improve diagnostic accuracy compared to standard MRI, particularly in the differential diagnosis between brain metastases and other intracranial lesions such as high-grade gliomas. Among these techniques, DCE-MRI provides quantitative information on vascular permeability and tumor microenvironment, while ASL offers complementary assessment of cerebral blood flow without the need for contrast agents. Beyond diagnosis, perfusion imaging enables longitudinal monitoring of tumor behavior, capturing dynamic changes in vascular characteristics over time, an aspect particularly relevant in patients who are not candidates for surgical biopsy. The integration of perfusion parameters into clinical workflows may therefore improve lesion characterization, support treatment stratification, and guide personalized follow-up strategies. However, substantial heterogeneity in acquisition protocols, parameter thresholds, and analytical methods limits reproducibility and inter-center comparability, highlighting the need for further standardization and prospective validation.

Within this framework, Senturk et al. [19] specifically investigated the role of DCE- and DSC-MRI in predicting treatment response after SRS in lung cancer brain metastases. Early post-treatment imaging (4–8 weeks) demonstrated that DCE-derived Ktrans outperformed DSC-derived parameters such as nCBV in identifying responders. Responding lesions showed significantly lower post-treatment Ktrans values and greater reductions from baseline, consistent with decreased vascular permeability and suppression of angiogenesis, whereas non-responding lesions exhibited stable or increased values, suggesting persistent vascular activity. Quantitatively, a Ktrans threshold of approximately 0.011 min^−1^ achieved high sensitivity for response prediction, although specificity remained limited, supporting its role primarily as a screening biomarker. The combination of DCE and DSC provided only marginal additional benefit.

Extending the role of DCE-MRI beyond treatment assessment, Young et al. [21] explored its potential for non-invasive molecular characterization in breast cancer brain metastases. The study demonstrated that Ktrans values were significantly higher in HER2-positive lesions, reflecting increased vascular permeability and a more angiogenic phenotype. A threshold of approximately 0.05 min^−1^ showed promising discriminative performance, supporting the use of DCE-derived parameters as imaging biomarkers of tumor molecular status. These findings are consistent with prior evidence of increased contrast enhancement and perfusion metrics (e.g., rCBV) in HER2-positive metastases, reinforcing the link between vascular permeability and tumor biology.

## 7. Translational Interpretation of DCE-MRI in Brain Metastases

Brain metastases represent biologically heterogeneous lesions in which angiogenesis, vascular permeability, and blood–brain barrier disruption play a central role. These processes underlie the imaging phenotype observed on MRI, where contrast enhancement reflects the degree of microvascular leakage and tumor–host interaction. Within this framework, DCE-MRI provides quantitative characterization of contrast-agent kinetics through parameters such as Ktrans, offering indirect information on microvascular permeability and linking imaging findings to underlying tumor biology. Increased permeability is typically associated with active tumor proliferation and angiogenesis, whereas treatment-related changes—such as radiation-induced injury and radionecrosis—reflect endothelial damage and altered vascular integrity, often resulting in different permeability dynamics.

## 8. Primary CNS Lymphoma

Ferazzoli et al. [22] investigated the complementary role of multiparametric MRI, including DCE, DSC, and diffusion kurtosis imaging (DKI), in the characterization of primary CNS lymphoma (PCNSL), a tumor that often mimics other brain lesions on conventional imaging. DCE-MRI provides information on vascular permeability and microvascular characteristics, showing increased Ktrans and Ve in PCNSL as a consequence of blood–brain barrier disruption. However, permeability metrics exhibit heterogeneous behavior: Kep demonstrated an inverse correlation with diffusion parameters (ADC and apparent diffusion coefficient derived from the kurtosis model (Dapp)), suggesting a relationship between increased cellularity and vascular permeability, whereas Ktrans did not show a consistent association, likely reflecting different underlying physiological processes. Perfusion imaging with DSC-MRI was consistent with known tumor biology, with PCNSL demonstrating lower rCBV compared to highly vascular tumors such as glioblastoma. Correlations among perfusion and permeability parameters were variable, with relCBF showing associations with Ktrans, Ve, and initial area under gadolinium curve, while the relationship between rCBV and Ktrans was weaker and less consistent, possibly due to technical and acquisition-related factors. A key contribution of the study is the integration of DKI, which demonstrated stronger correlations with both DCE- and DSC-derived parameters compared to conventional diffusion imaging. In particular, Dapp correlated with Kep and apparent diffusional kurtosis with rCBV, suggesting that DKI provides a more sensitive representation of tumor heterogeneity and microstructural complexity. Overall, these findings support the use of a multiparametric MRI approach in PCNSL, in which DCE contributes information on vascular permeability, DSC on perfusion, and DKI on tissue heterogeneity, offering complementary insights into tumor biology. However, limitations such as small sample size and lack of standardized protocols may affect reproducibility, highlighting the need for further validation.

## 9. DCE-MRI and Artificial Intelligence: Toward Integrated and Reproducible Imaging Biomarkers

Artificial intelligence is increasingly integrated into DCE-MRI across the entire imaging pipeline, addressing key limitations related to data quality, parameter estimation, and biological interpretation [23,24,25]. At the acquisition and preprocessing level, deep learning-based approaches improve image quality through denoising and super-resolution, enhancing signal stability and the reliability of pharmacokinetic modeling. Similarly, automated segmentation techniques reduce operator dependence and minimize bias, particularly in the exclusion of large vascular structures that may confound permeability measurements [23,24]. At the quantitative level, AI-driven modeling approaches overcome intrinsic limitations of conventional pharmacokinetic analysis, such as dependence on arterial input function estimation and sensitivity to noise. Deep learning frameworks enable more robust and efficient extraction of permeability-related parameters, improving reproducibility while substantially reducing computational burden. In parallel, machine learning-based methods highlight how analytical choices—such as vascular input function selection—directly influence parameter accuracy and diagnostic performance. Beyond parameter estimation, the integration of DCE-MRI with radiomics and deep learning enables a higher-level characterization of tumor biology [25,26]. By combining permeability metrics with diffusion and structural imaging features, these approaches capture intratumoral heterogeneity and support non-invasive molecular stratification, reinforcing the role of DCE-MRI as a bridge between imaging phenotype and underlying tumor biology. Advanced architectures, including transformer-based models, further enhance the ability to extract biologically meaningful patterns, supporting the concept of imaging-derived “virtual biopsy” [27,28]. From a clinical perspective, the synergy between DCE-MRI and artificial intelligence lies in the transition from descriptive imaging to quantitative, reproducible, and biologically interpretable biomarkers. However, despite these advances, clinical implementation remains limited by the need for standardized acquisition protocols, large multicenter validation datasets, and improved model interpretability. As such, the integration of AI with DCE-MRI represents a critical step toward precision neuro-oncology but requires further validation to ensure robustness, generalizability, and clinical applicability.

## 10. Integrated Overview of DCE-MRI Across Neuro-Oncologic Settings

To provide an integrated overview before the Discussion, the main biological mechanisms, DCE-MRI patterns, and clinical applications across neuro-oncologic settings are summarized in Table 4.

## 11. Discussion

Differentiating post-radiation necrosis (PRN) from tumor recurrence remains a major challenge in neuro-oncology, due to the substantial overlap in imaging features and shared pathophysiological effects of radiotherapy. Conventional MRI is often insufficient, as both conditions typically present with contrast enhancement and perilesional edema, leading to diagnostic uncertainty and potential misclassification [9]. Beyond conventional contrast-enhanced MRI, DCE-MRI provides quantitative information on contrast kinetics and blood–brain barrier disruption, thereby enabling further characterization of suspicious enhancing lesions in the post-treatment setting (Amer). This additional information may support the differentiation of treatment-related changes from viable tumor, although substantial overlap between these conditions prevents DCE-derived parameters from being interpreted as standalone diagnostic biomarkers. In this context, advanced imaging techniques have significantly improved diagnostic assessment by providing complementary biological information. Perfusion MRI provides complementary information through DCE- and DSC-MRI. Whereas DSC-MRI primarily characterizes tissue perfusion and relative cerebral blood volume, DCE-MRI provides additional information on contrast-agent leakage and microvascular permeability through pharmacokinetic parameters such as Ktrans and Ve. Thus, DCE-MRI should be regarded as complementary to, rather than a replacement for, DSC-MRI in the assessment of treatment-related changes and tumor progression [11,22]. Regarding reproducibility, no single quantitative DCE-derived metric can currently be considered universally robust across institutions and acquisition protocols. Although parameters such as Ktrans, Vp, and AUC have shown relatively favorable reproducibility in individual studies [16], their absolute values remain sensitive to acquisition settings, arterial input function estimation, pharmacokinetic modeling, and post-processing choices. Consequently, relative or multiparametric interpretation may currently be more transferable than the application of fixed quantitative thresholds across centers. Conversely, several more advanced applications of DCE-MRI remain predominantly investigational. These include voxel-wise and habitat-based analyses of intratumoral heterogeneity, imaging-based molecular stratification, and the use of fixed quantitative cutoffs derived from individual cohorts. Although these approaches have shown promising associations with tumor biology, molecular features, and treatment response, their clinical translation is currently limited by small or single-center cohorts, methodological heterogeneity, and insufficient prospective external validation [14,15,21]. Similarly, PET imaging contributes metabolic insights. In particular, [18F]-Fluciclovine PET/MRI has demonstrated high diagnostic accuracy in differentiating true glioblastoma progression from treatment-related changes, with consistent SUVmax thresholds across two institutions, supporting its reproducibility and highlighting its complementary role within a multiparametric imaging framework [29]. However, despite these advances, no single imaging modality consistently achieves optimal sensitivity and specificity. Variability in acquisition protocols, analytical methods, and reported thresholds limits reproducibility and clinical standardization. Consequently, there is increasing consensus that a multiparametric approach, integrating structural, perfusion, diffusion, and metabolic imaging, represents the most reliable strategy for differentiating PRN from recurrence. From a clinical standpoint, this distinction is critical, as misclassification may lead either to delayed treatment in the case of recurrence or to unnecessary interventions in patients with PRN. Nevertheless, current evidence remains limited by heterogeneity and the predominance of retrospective studies, underscoring the need for prospective validation and standardized imaging protocols. Overall, while advanced imaging—particularly perfusion MRI and PET—has substantially improved non-invasive characterization, multimodal integration remains essential, and further efforts are required to enhance reproducibility and clinical applicability. An emerging application of DCE-MRI is the evaluation of non-enhancing peritumoral areas (NEPAs), which are poorly characterized by conventional imaging. As highlighted by Scola et al. [30], although permeability metrics such as Ktrans may not significantly differ due to a relatively preserved blood–brain barrier, subtle increases in Ktrans and Vp may indicate infiltrative tumor components with early microvascular alterations. These findings suggest that DCE-MRI can capture biologically relevant processes beyond contrast-enhancing tumor margins, with potential implications for tumor delineation and treatment planning. However, evidence remains limited, and the interpretation of permeability parameters in non-enhancing tissue is not yet standardized. Recent methodological advances aim to overcome key limitations of multiparametric perfusion imaging. In this context, Sanvito et al. [31] proposed a dual-echo DSC approach enabling simultaneous assessment of perfusion and permeability within a single acquisition, reducing the need for separate sequences and multiple contrast administrations. This technique extracts both T2*- and T1-related signals from a single dynamic acquisition, allowing for indirect estimation of permeability while preserving perfusion information. Novel metrics, such as TRATE and ΔR1,ss, may provide more robust indicators of leakage effects and blood–brain barrier disruption. From a broader perspective, these developments are highly relevant for the clinical application of DCE-derived biomarkers. One of the main limitations of DCE-MRI remains the lack of standardization and the variability introduced by acquisition and modeling choices. Techniques that integrate perfusion and permeability assessment while reducing technical complexity may therefore improve reproducibility and facilitate wider clinical adoption. However, current evidence remains preliminary, with limited validation in small cohorts. As such, although dual-echo approaches represent a promising step toward more efficient and standardized imaging, their real-world applicability and diagnostic impact require further prospective validation. Emerging computational approaches may further support the standardization and clinical translation of DCE-MRI, although their role remains investigational. Deep learning-based methods have been explored for image denoising and super-resolution, with the aim of improving the reliability of DCE-derived parameters and arterial input function estimation [32], while automated vascular segmentation may reduce bias related to large-vessel contamination [25]. More recent approaches have investigated direct or accelerated estimation of pharmacokinetic parameters, potentially reducing dependence on conventional post-processing pipelines [24,33]. However, these methods remain limited by training-data dependence, methodological heterogeneity, and insufficient external and prospective validation. Accordingly, artificial intelligence should currently be regarded as a promising methodological tool for improving DCE-MRI processing and reproducibility rather than as an established component of routine neuro-oncologic decision-making. Optimal management of patients with brain metastases requires accurate detection at diagnosis and reliable differentiation between treatment-related effects and tumor progression during follow-up. While contrast-enhanced MRI remains the standard imaging modality, advanced techniques have improved the characterization of tumor biology. Among these, DCE-MRI provides quantitative information on vascular permeability through pharmacokinetic parameters such as Ktrans and Vp, contributing to the differentiation of recurrent brain metastases from treatment-related effects [34]. DCE-MRI is sensitive to treatment-induced vascular changes and may provide complementary information for distinguishing viable tumor from treatment-related effects in the evaluation of brain metastases. Additional modalities, including diffusion MRI, MR spectroscopy, and PET imaging, provide complementary information, while artificial intelligence approaches may further enhance diagnostic performance. An important tension in the current DCE-MRI literature concerns the gap between theoretical potential and clinical feasibility. Although quantitative permeability metrics are biologically grounded and conceptually attractive, their practical use is hindered by substantial variability across scanners, protocols, and modeling approaches. Even within the same patient, repeated examinations may yield inconsistent values, making published cutoff thresholds difficult to reproduce. Overall, current evidence supports a multiparametric imaging approach in which DCE-MRI provides complementary information for assessing disease status and treatment response in brain metastases.

## 12. Limitations

A critical appraisal of the current evidence on DCE-MRI requires balancing its reported diagnostic performance with the underlying methodological limitations of the available studies. A substantial proportion of the available evidence is based on retrospective analyses, often conducted in single-center settings with relatively small and heterogeneous patient cohorts. These factors may limit the generalizability of the findings. Another major limitation concerns the lack of standardization across studies, particularly regarding DCE-MRI acquisition protocols, pharmacokinetic modeling, parameter thresholds, and post-processing methods. This variability complicates direct comparison between studies and limits the reproducibility and clinical translation of quantitative imaging biomarkers such as Ktrans, Vp, and Ve. These factors make quantitative thresholds difficult to generalize and have contributed to the widespread clinical preference for qualitative curve analysis. This review is narrative in nature and reflects these methodological constraints, which currently limit the translational reliability of quantitative DCE-MRI. In addition, several studies relied on imaging-based reference standards rather than histopathological confirmation, particularly in the differentiation between tumor progression and treatment-related changes. This may introduce diagnostic uncertainty and affect the accuracy of reported performance metrics. Technical limitations inherent to DCE-MRI, including sensitivity to noise, variability in arterial input function estimation, and partial volume effects, were also frequently reported. Although recent advances in deep learning and automated processing aim to address these issues, their clinical implementation remains limited and requires further validation. Finally, emerging approaches such as radiomics and artificial intelligence, while promising, are still constrained by limited external validation, potential overfitting, and the lack of interpretability, which may hinder their integration into routine clinical practice.

## 13. Conclusions

Differentiating tumor progression from treatment-related alterations remains a major challenge in neuro-oncology, particularly in the post-treatment setting where conventional MRI lacks specificity. In this context, DCE-MRI provides biologically grounded information on contrast-agent kinetics, offering indirect markers of microvascular permeability and blood–brain barrier integrity that may support the distinction between these entities. Its clinical value is most established in gliomas and is increasingly being investigated in brain metastases and primary CNS lymphoma. However, DCE-MRI remains less universally implemented in routine neuro-oncologic practice than conventional MRI, diffusion-weighted imaging, or DSC perfusion, partly because of its greater technical complexity and the limited standardization of acquisition and post-processing methods. Accordingly, DCE-MRI should not be regarded as a standalone diagnostic technique but rather as a complementary component of multiparametric imaging assessment, integrating permeability information with structural, diffusion, and perfusion biomarkers. Further protocol harmonization and large prospective multicenter studies are required before quantitative DCE-derived biomarkers can achieve widespread clinical implementation.

## Figures and Tables

**Table 1:** Overview of the biological mechanisms, DCE-MRI permeability patterns, complementary imaging features, and clinical implications for pseudoprogression, radiation necrosis, and true tumor progression, highlighting how vascular injury, inflammation, and active angiogenesis shape diagnostic interpretation in the post-treatment setting.

Entity	Biological Background	DCE-MRI Pattern/Parameters	Additional Imaging Features	Clinical Implications	Time of Onset	Key Interpretation
Pseudoprogression (PsP)	Treatment-induced inflammation, BBB disruption, immune activation; absence of true tumor proliferation	Lower vascular activity; relatively lower Ktrans compared to progression; variable permeability patterns	Increased T2/FLAIR signal (edema); may show transient contrast enhancement	Usually self-limiting; associated with favorable treatment response; requires longitudinal assessment	- Early (3–6 months) - Later presentations are increasingly recognized and may vary according to treatment context - Often associated with favorable treatment response	Reflects treatment response rather than true progression
Radiation necrosis (RN)	Endothelial damage, microvascular thrombosis, chronic hypoxia, VEGF-mediated abnormal angiogenesis, tissue necrosis	Altered permeability due to BBB disruption; heterogeneous Ktrans; no reliable thresholds	Ring enhancement; “Swiss cheese” pattern; extensive edema; possible mass effect	Irreversible tissue injury; may require corticosteroids or surgical management	- Late (3–40 months or later) - Irreversible tissue damage	Represents late radiation-induced tissue damage
Tumor progression (TP)	Active tumor proliferation, angiogenesis, increased microvascular density, persistent BBB disruption	Increased Ktrans, Ve; higher peak enhancement; rapid contrast kinetics	Progressive contrast enhancement; lower ADC (higher cellularity); elevated rCBV	Requires treatment escalation or change in therapy	Variable; may occur at different times during post-treatment follow-up	Indicates active tumor growth and disease progression

**Table 2:** Overview of key DCE-MRI studies in high-grade gliomas, summarizing permeability metrics and combined multiparametric approaches used to distinguish treatment-related changes, predict progression, assess heterogeneity, and support molecular stratification. Abbreviations: ADC, apparent diffusion coefficient; AUC, area under the curve; DCE, dynamic contrast-enhanced; DTI, diffusion tensor imaging; GBM, glioblastoma; HGG, high-grade glioma; iAUC, initial area under the curve; MIS4, Multimodal Imaging Subregion 4; NMD, nonmeasurable disease; NEPTR, non-enhancing peritumoral region; PD, progressive disease; PE%, peak enhancement percentage; PsP, pseudoprogression; rCBV, relative cerebral blood volume; TR, treatment-related changes; TRC, treatment-related changes; TP, tumor progression.

Study	Population	Clinical Setting	DCE Parameters	Main Findings	Key Contribution
[10]	GBM post-RT	TRC vs. TP	PE%, iAUC	PE% highly accurate (AUC 0.94); lower in TRC	Semiquantitative DCE as practical diagnostic tool
[11]	GBM post-RT	PD vs. PsP	Ktrans, Vp, Ve + rCBV	Combined DCE + DSC improves accuracy; thresholds identified	Multiparametric integration improves early diagnosis
[12]	High-grade gliomas	TP vs. TR (incl. NEPTR)	Ktrans, Ve, iAUC + FA/ADC	Higher permeability + lower diffusion in TP; high AUC (0.983)	Integration of DCE + DTI; peritumoral analysis
[13]	HGG (NMD)	Early progression prediction	Ktrans, Ve (histogram)	High predictive accuracy (AUC up to 0.927)	Histogram analysis captures tumor heterogeneity
[14]	Diffuse gliomas	Molecular stratification	Ktrans + ADC (habitat)	Distinct habitats correlate with IDH status and prognosis	Spatial heterogeneity mapping (habitat imaging)
[15]	Recurrent GBM	Heterogeneity/treatment response	Ktrans + diffusion	Identification of resistant subregions (MIS4)	Imaging-based tumor phenotyping
[16]	HGG post-treatment	Reliability study	AUC, Vp, Ktrans	Quantitative metrics more reproducible than qualitative	Supports quantitative imaging approach

**Table 3:** Summary of key DCE-MRI parameters and clinical applications across studies on brain metastases. The table highlights how permeability metrics and diffusion measures contribute to tumor characterization, treatment-response assessment, and molecular stratification while underscoring limitations related to cohort heterogeneity, retrospective designs, and the need for broader validation.

Study	Population	Clinical Setting	DCE Parameters	Main Findings	Key Contribution	Limitations
[17]	Mixed brain metastases	Imaging characterization	Ktrans, ADC	ADC inversely correlates with tumor cellularity; DCE reflects vascular permeability	Integration of diffusion and permeability for tumor characterization	Limited specificity; heterogeneous cohort
[18]	Brain metastases	Treatment response	Ktrans	Reduction in Ktrans associated with treatment response; stability/increase in non-responders	Longitudinal assessment of vascular permeability as response biomarker	Heterogeneous population; need for validation
[19]	Lung cancer brain metastases	Post-SRS response prediction	Ktrans, nCBV	Lower post-treatment Ktrans in responders; threshold ~0.011 min^−1^	Early prediction of treatment response using DCE-MRI	Retrospective design; limited specificity
[20]	Systematic review	Multiple settings	Ktrans, rCBV	DCE and DSC improve diagnostic accuracy vs. conventional MRI	Evidence synthesis supporting multiparametric imaging	High heterogeneity in protocols and thresholds
[21]	Breast cancer brain metastases	Molecular characterization	Ktrans	Higher Ktrans in HER2-positive lesions	Non-invasive molecular stratification using DCE-MRI	Small sample size; single-center study

**Table 4:** Overview of key biological mechanisms, DCE-MRI permeability patterns, and clinical roles across major post-treatment brain entities, highlighting how vascular injury, inflammation, and tumor-driven angiogenesis shape quantitative parameters used for differential diagnosis.

Setting	Biological Background	DCE-MRI Pattern/Parameters	Clinical Role	Key References
Radiation necrosis	Endothelial damage, microvascular thrombosis, hypoxia, HIF-1α activation, VEGF-mediated abnormal angiogenesis, inflammatory cascade	Altered permeability related to BBB disruption; no standardized thresholds	Differentiation from tumor recurrence remains challenging; requires multiparametric integration	[6,7,8]
PsP	Treatment-induced inflammatory infiltration; immune activation; transient BBB disruption	Lower vascular activity compared to progression; overlapping permeability patterns	Avoid misclassification and inappropriate treatment changes; requires longitudinal and clinical correlation	[4,5]
Tumor progression (gliomas)	Active angiogenesis, increased microvascular density, BBB disruption	Increased Ktrans, Ve; higher peak enhancement; rapid enhancement kinetics	Differentiation from treatment-related changes; early detection of progression	[10,13]
Glioblastoma (post-treatment)	Coexistence of viable tumor, necrosis, and inflammation; marked intratumoral heterogeneity	Higher Ktrans and rCBV in progression; lower values in treatment-related changes; spatial heterogeneity captured by advanced analysis	Improved diagnostic accuracy using multiparametric approaches (DCE + diffusion/perfusion)	[11,12,14,15]
Brain metastases	Angiogenesis and BBB disruption in tumor; endothelial damage and vascular injury in treatment-related effects	Ktrans reflects vascular permeability; reduction after treatment response; stability/increase in non-responders	Treatment response assessment; differentiation between viable tumor and treatment-related effects	[18,19]
Primary CNS lymphoma (PCNSL)	BBB disruption with high cellularity; distinct vascular profile compared to glioblastoma	Increased Ktrans and Ve; variable correlation with other parameters; heterogeneous behavior of permeability metrics	Characterization and differentiation from other tumors within multiparametric framework	[22]

## Data Availability

The original contributions presented in this study are included in the article. Further inquiries can be directed to the corresponding author.
